# Role of Oxidative, Nitrative, and Chlorinative Protein Modifications in Aging and Age-Related Diseases

**DOI:** 10.1155/2018/3267898

**Published:** 2018-08-07

**Authors:** Izabela Sadowska-Bartosz, Grzegorz Bartosz, Tilman Grune, Jolanta Sereikaite

**Affiliations:** ^1^Department of Analytical Biochemistry, Faculty of Biology and Agriculture, University of Rzeszow, Zelwerowicza Street 4, 35-601 Rzeszow, Poland; ^2^Department of Molecular Biology, Faculty of Biology and Environmental Protection, University of Łódź, Pomorska 141/143, 90-236 Łódź, Poland; ^3^Department of Molecular Toxicology, German Institute of Human Nutrition Potsdam-Rehbruecke (DIfE), Arthur-Scheunert-Allee 114-116, 14558 Nuthetal, Germany; ^4^Department of Chemistry and Bioengineering, Faculty of Fundamental Sciences, Vilnius Gediminas Technical University, Sauletekio al. 11, LT-2040 Vilnius, Lithuania

Despite extensive studies, the molecular basis of physiological aging is still poorly understood. Reactive oxygen species (ROS) and reactive nitrogen species (RNS) as well as reactive halogen species (RXS) species are believed to play a key role in the aging process. They are generated during aerobic metabolism in living organisms. The term “*reactive oxygen species*” includes both free radicals (molecules having an odd electron, like superoxide radical anion (O_2_^•−^) and hydroxyl radical (HO^•^)) and species that are not free radicals, like hydrogen peroxide (H_2_O_2_), singlet oxygen (^1^O_2_), and ozone (O_3_). The primary source of RNS is usually the nitric oxide radical (^•^NO). In consequence of ROS and RNS reactions, peroxynitrite ONOO^−^, anion of peroxynitrous acid ONOOH, may be formed via the near diffusion-limited reaction of ^•^NO and O_2_^•−^. The term “*reactive nitrogen species*” includes also nitrous acid (HNO_2_), dinitrogen trioxide (N_2_O_3_), nitrosyl anion (NO^−^), nitrosyl cation (NO^+^), nitrogen dioxide radical (^•^NO_2_), peroxynitrate (ONOOO^−^), peroxynitric acid (ONOOOH), nitryl chloride (NO_2_Cl), and nitronium cation (NO_2_^+^) [1, 2]. Reactive halogen species include HOCl, HOBr, HOI, chlorine, bromine, iodine, and so on. Hypohalogenous acids (HOX; X=F, Cl, Br, or I) are formed in the body mainly by oxidation of halogen ions by myeloperoxidase. The imbalance between ROS, RNS, and RXS production and the antioxidant defense, in favor of prooxidants, is called oxidative, nitr(os)ative, and halogenative stress (OS, NS, and XS), respectively. Although at physiological concentrations ROS, RNS, and RXS can function as signaling molecules regulating cell proliferation, growth, differentiation, and apoptosis [3, 4], they react with and damage all classes of endogenous macromolecules including proteins, nucleic acids, lipids, and carbohydrates [5]. Proteins are the main targets for such modifications as they are the most abundant cell components in the terms of mass content. The level of protein damage increases under stress conditions and can be in principle an integrative measure of the exposure to OS, NS, and XS. Another source of protein modification is glycoxidation leading to the formation of advanced glycation end products (AGEs).

In this issue, the comprehensive review by A. L. Santos and A. B. Lindner presents the interplay of nonenzymatic posttranslational protein modifications in aging-associated molecular processes underlying eukaryotic aging. Understanding of the roles played by posttranslational protein modifications in aging and age-related diseases can facilitate targeted therapies or interventions in these diseases and the aging process itself. The review highlights also the potential of simple prokaryotic models to uncover complex aging-associated molecular processes in the emerging field of microbiogerontology. D. Weber et al. summarize the results of the European multicenter study MARK-AGE from 1559 participants. They demonstrate that, among others, protein carbonyls and 3-nitrotyrosine are biomarkers with the highest correlation with age.

Protein carbonyls are the most frequently assayed protein modification by ROS, RNS, and RXS. In this issue, M. Adamczyk-Sowa et al. demonstrate that blood serum proteins of multiple sclerosis patients suffer oxidative modifications, which are attenuated by interferon beta and further by coadministration of interferon beta and the antioxidant melatonin.

The pyrin domain-containing 3 (NLRP3) inflammasome, as a vital component of the innate immune system, is implicated in the pathogenesis of type 2 diabetes. X. Kong et al. show that the administration of AGEs (120 mg/kg for 6 weeks) in C57BL/6J mice induced an abnormal response to glucose, pancreatic *β*-cell ultrastructural lesion, and cell death. Ablation of the NLRP3 inflammasome or treatment with antioxidant N-acetyl-cysteine (NAC) clearly ameliorated these effects, suggesting AGE-induced NLRP3 inflammasome activation as a mechanism for pancreatic islet damage. J. Bai et al. provide evidence that ghrelin, a growth hormone-releasing peptide, protects against H_2_O_2_-induced oxidative stress in human lens epithelial cells and rat lenses. Their results suggest that ghrelin may prevent the progression of cataracts, a result that is potentially valuable for the clinical treatment of cataracts.

The articles contained in this special issue extend our knowledge on the role of nonenzymatic posttranslational protein modifications in age-related diseases and aging.

*Izabela Sadowska-Bartosz* *Grzegorz Bartosz* *Tilman Grune* *Jolanta Sereikaite*
